# Synthesis, Characterization and Application of Polypyrrole Functionalized Nanocellulose for the Removal of Cr(VI) from Aqueous Solution

**DOI:** 10.3390/polym13213691

**Published:** 2021-10-26

**Authors:** Norah Salem Alsaiari, Khadijah Mohammedsaleh Katubi, Fatimah Mohammed Alzahrani, Abdelfattah Amari, Haitham Osman, Faouzi Ben Rebah, Mohamed A. Tahoon

**Affiliations:** 1 Chemistry Department, College of Science, Princess Nourah Bint Abdulrahman University, Riyadh 11671, Saudi Arabia; nsalsaiari@pnu.edu.sa; 2 Department of Chemical Engineering, College of Engineering, King Khalid University, Abha 61411, Saudi Arabia; haman@kku.edu.sa; 3 Research Laboratory of Energy and Environment, Department of Chemical Engineering, National School of Engineers, Gabes University, Gabes 6072, Tunisia; 4 Higher Institute of Biotechnology of Sfax (ISBS), Sfax University, P.O. Box 263, Sfax 3000, Tunisia; benrebahf@yahoo.fr; 5 Department of Chemistry, College of Science, King Khalid University, P.O. Box 9004, Abha 61413, Saudi Arabia; tahooon_87@yahoo.com; 6 Chemistry Department, Faculty of Science, Mansoura University, Mansoura 35516, Egypt

**Keywords:** adsorption, cellulose, polypyrrole, nanomaterials, Cr(VI), water treatment

## Abstract

Heavy metals are toxic substances that pose a real danger to humans and organisms, even at low concentration. Therefore, there is an urgent need to remove heavy metals. Herein, the nanocellulose (NC) was synthesized by the hydrolysis of cellulose using sulfuric acid, and then functionalized using polypyrrole (ppy) through a polymerization reaction to produce polypyrrole/nanocellulose (ppy/NC) nanocomposite. The synthesized nanocomposite was characterized using familiar techniques including XRD, FT-IR, SEM, TEM, and TGA. The obtained results showed a well-constructed nanocomposite with excellent thermal stability in the nano-sized scale. The adsorption experiments showed that the ppy/NC nanocomposite was able to adsorb hexavalent chromium (Cr(VI)). The optimum pH for the removal of the heavy metal was pH 2. The interfering ions showed minor effect on the adsorption of Cr(VI) resulted from the competition between ions for the adsorption sites. The adsorption kinetics were studied using pseudo 1st order and pseudo 2nd order models indicating that the pseudo second order model showed the best fit to the experimental data, signifying that the adsorption process is controlled by the chemisorption mechanism. Additionally, the nanocomposite showed a maximum adsorption capacity of 560 mg/g according to Langmuir isotherm. The study of the removal mechanism showed that Cr(VI) ions were removed via the reduction of high toxic Cr(VI) to lower toxic Cr(III) and the electrostatic attraction between protonated ppy and Cr(VI). Interestingly, the ppy/NC nanocomposite was reused for Cr(VI) uptake up to six cycles showing excellent regeneration results. Subsequently, Cr(VI) ions can be effectively removed from aqueous solution using the synthesized nanocomposite as reusable and cost-effective adsorbent.

## 1. Introduction

Heavy metals pollution is becoming a serious environmental problem around the world [1]. Various toxic metals can cause serious problems to human and marine organisms [2]. Among these metals, chromium (Cr) is considered to be one of the most dangerous metals. Cr naturally exists in two oxidation states Cr(VI) and Cr(III) [3]. The two oxidation states have different mobilities and poisonousness. Cr(III) is stated to be 500 times less poisonous than Cr(VI) [4]. At low quantities, Cr(III) is less hazardous micronutrient and is required for body metabolism [5]. In contrast, Cr(VI) exhibits a variety of toxic, mutagenic, and carcinogenic effects to humans. It is expected to cause very dangerous chromosomal mutations by modifying DNA transcription steps [6]. Additionally, Cr(VI) ions can cause gastric, liver, and kidney damage as well as lung cancer [7].

Generally, photography, textile industries, fertilizers production, wood preservation, chrome coating, leather tanning, stainless steel production, and pigment manufacturing are the main sources of Cr(VI) ions [8]. Thus, the treatment of wastewater generated by these industries is crucial to prevent pollution to make the environment safe and clean. In recent years, the removal of Cr (VI) ions from wastewaters before their release into the environment has been considered as a major challenge facing scientists. The treatment strategy aims to reduce Cr(VI) concentrations from water/wastewater to acceptable levels. According to the World Health Organization (WHO), the permitted level of Cr(VI) ions is 50.0 µg/L for drinking water is 50.0 µg/L [9], while this level is 5.0 µg/L and 200.0 µg/L for underground water and industrial wastewater, respectively, according to European Union [10].

The removal of Cr(VI) ions from aqueous solutions was achieved using different techniques such as membrane separation, reverse osmosis, solvent extraction, ion exchange, reduction, chemical and electrochemical precipitations, dialysis, and adsorption [11,12,13,14,15,16]. Among all methods, adsorption is widely used for the removal of Cr(VI) ions from aqueous solutions due to many reasons such as ease regeneration of adsorbents, high efficiency, simple operation, and cost-effectiveness [17]. However, the using of conventional adsorbents for the removal of Cr (VI) ions face many problems such as insufficient hydrophilic surface and functional groups that led to secondary contamination besides the low adsorption capacity due to the low surface area [18]. These limitations of conventional adsorbents can be overcome by using nanomaterials (NMs) as adsorbents. NMs have exceptional properties such as high surface area providing high capacity for metal capturing, ease synthesis, simple functionalization, and low cost [19]. However, the using of NMs as adsorbents for the removal of metals from aqueous solutions was associated with poisonous problems [20]. So, eco-friendly NMs such as nanostructured polysaccharide have been recently examined as friendly adsorbents for the removal of metals from aqueous solutions [21,22]. Interestingly, cellulose is one of the most attractive materials useful as adsorbents to remove organic and inorganic pollutants from wastewater. In this context, the developments of the use of nanocellulose for various applications in environmental remediation, such as its use as adsorbent for enhanced and selective capturing of metallic ions, are well documented in the literature [23,24,25]. However, the breakdown and agglomeration of cellulose in water limits its use as adsorbent. This limitation can be overcome by the chemical modification of cellulose using polymeric material via the OH groups. This chemical modification of cellulose helps in increasing the adsorption capacity of nanocellulose besides the increasing of adsorbent mechanical strength [26]. Among various polymers, polypyrrole (ppy) has received considerable interest because of its interesting properties (excellent chemical stability, conductivity, biocompatibility, low oxidation potential, easy synthesis, low cost, etc.) and its good compatibility with different nanoparticles. The use of ppy to adsorb pollutants has been reported as adsorbent for the removal of metals. Additionally, ppy was used for the modification of magnetic Fe_3_O_4_/SiO_2_ nanocomposite for the enhanced adsorption of Congo red dye and Cr(VI) ions [27]. However, its use in large scale wastewater treatment processes is limited by its poor processability and lack of mechanical properties. Therefore, the recorded disadvantages can be eliminated by compositing ppy with other materials (biological materials, chemical polymers, agro-industrial wastes, etc.). Interestingly, the ppy is very suitable for the functionalization of nanocellulose due to good ion exchange performance and high chemical stability [28].

Herein, the nanocellulose was synthesized via the hydrolysis of cellulose using sulfuric acid followed by the functionalization of nanocellulose by the ppy polymer to form the nanocomposite (ppy/NC). The ppy/NC nanocomposite was characterized using different techniques and examined for the removal of Cr(VI) ions from water.

## 2. Materials and Methods

### 2.1. Chemicals

Cellulose with high purity was supplied from HiMedia. Sulfuric acid (H_2_SO_4_), ammonium persulfate ((NH_4_)_2_S_2_O_8_), and pyrrole were supplied from Aladdin (Shanghai, China). Hydrochloric acid (HCl) was supplied from Winlab Chemical (India). Potassium dichromate (K_2_Cr_2_O_7_) was supplied from Sigma-Aldrich (Schnelldorf, Germany). All chemicals were used as received without any modification. Distilled water was used for the preparation of all solutions.

### 2.2. Nanocellulose Synthesis

Nanocellulose (NC) was synthesized as described in the literature [29] with some modifications. Firstly, a colloidal solution of cellulose was prepared by suspending 20.0 g of cellulose powder in water followed by hydrolysis process in which the colloidal solution was treated by 60 mL of 65.0% sulfuric acid with continuous stirring for 4.0 h at a temperature of 40 °C. Then, the suspension was centrifuged for 10 min at 8000× *g* rpm followed by dialysis to remove the excess of acid. Then, the suspension was dialyzed against deionized water using membranes of 12,000–14,000 Da molecular weight cutoff until the neutral pH value was achieved. The obtained suspension was finally ultra-sonicated for 45 min in an ice bath then stored at a temperature of 4 °C. Subsequently, the suspension was ready to be used in the next step.

### 2.3. Synthesis of Nanocellulose/Polypyrrole Nanocomposite

Firstly, the polypyrrole was synthesized via the polymerization of pyrrole (1.35 mL of pyrrole in 200 mL of water) by using (NH_4_)_2_S_2_O_8_ (40.0 mL of 0.15 M) with continuous stirring until the color changed from white to black. The pyrrole was mixed with the suspension solution of nanocellulose under sonication process for 40 min at 3 °C using a tip sonicator at a fixed power of 30 W. Then, the mixture was reserved for half-hour at a temperature of 3.0 °C followed by the removal of the top layer from the mixture. Finally, the lyophilization at −80 °C and 20 Pa besides the solvent exchange with *t*-butanol was used to get the powder of ppy/NC nanocomposite. The obtained nanocomposite was stored at room temperature until use for characterization and application.

### 2.4. Adsorption Experiments

The adsorption experiments were conducted by mixing the solution of Cr(VI) with the adsorbent at certain conditions of initial concentration, adsorbent dosage, contact time, and pH in shaker at 200 rpm. After each adsorption experiment, the adsorbent was separated using a 0.45 µm filter and the remaining solution was examined for the presence of Cr(VI) ions using UV spectrophotometer (λ_max_ = 540 nm) after a complex formation between chromium ions and 1,5-diphenylcarbazide. To study the pH effect on the adsorption, 60 mL of Cr(VI) solution (100 mg/L) was mixed with 15 mg of adsorbent at different pH values, ranging from 2.0 to 8.0. Additionally, the removal efficiency of Cr(VI) ions was determined in the presence of competing ions (NaCl, Ca^2+^, Zn^2+^, CO_3_^2−^, PO_4_^3−^, and SO_4_^2−^) added at 0.1 M. To study the adsorption kinetics, 300 mL of Cr(VI) ions solutions with different initial concentrations ranged from 100 mg/L to 200 mg/L were mixed with 75 mg of adsorbent at a pH of 2. To study the adsorption isotherm, Cr(VI) ions solutions with different initial concentrations ranged from 50 mg/L to 300 mg/L were mixed with the adsorbent at pH of 2. The adsorption capacity (q_e_) of the adsorbent was calculated using Equation (1).

q_e_ = ((C_o_ − C_e_)/m) × V, (1)

where C_o_, C_e_, m, and V denote the initial concentration (mg/L), equilibrium concentration (mg/L), the mass of adsorbent (g), and volume of the solution, respectively. The removal percentage (%) was calculated using Equation (2).

Removal (%) = (C_o_ − C_e_/C_o_) × 100 (2)

Moreover, the reusability of ppy/NC nanocomposite for the removal of Cr(VI) ions was studied up to five successive cycles using 1 M HCl as eluent. In each cycle, 60 mL of metallic ions solution was mixed with 15.0 mg of the adsorbent and agitated for 1 day followed by the collection of the adsorbent. Then, the adsorbent was regenerated using the eluent solution for 1.0 h. After that, the adsorbent was washed using water until neutral pH and subsequently, the adsorbent was ready for the next adsorption–desorption cycle. For errors determination, all experiments were done triplicate.

## 3. Results and Discussion

### 3.1. The Characterization of ppy/NC Nanocomposite

**Figure 1:**
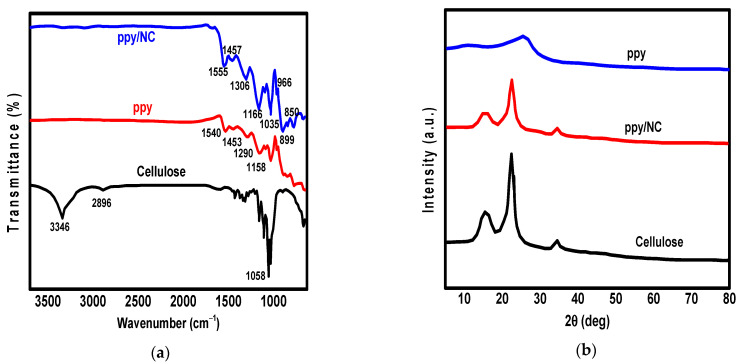

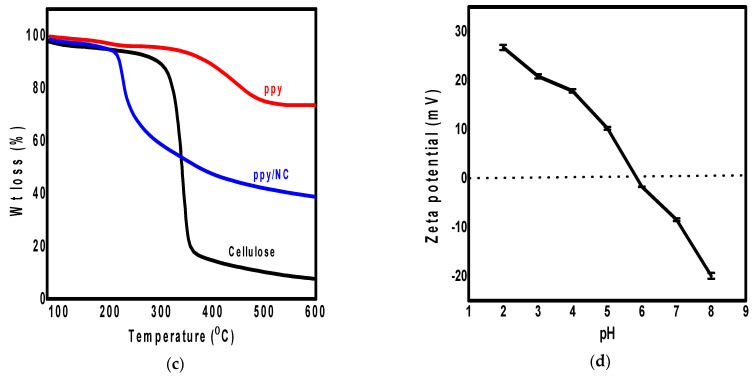
FT-IR (**a**) XRD (**b**), TGA (**c**), and zeta potential at various pH (**d**) of the synthesized ppy, cellulose, and ppy/NC nanocomposite.

The synthesized materials including NC, ppy, and ppy/NC nanocomposite were characterized using the familiar techniques (FT-IR, XRD, TGA, SEM, and TEM). For the determination of functional groups, FT-IR spectra were shown in Figure 1a. According to Figure 1a, the synthesized NC showed the characteristic cellulose absorption bands like the peaks at 1058 cm^−1^, 2896 cm^−1^, and 3346 cm^−1^ that are attributed to the stretching vibrations of C–O–C, CH_2_, and OH, respectively [30].

However, the FT-IR of ppy/NC nanocomposite showed only the appearance of ppy representative peaks like band of N–H wagging at 850 cm^−1^, C–H in-plane bending at 966 cm^−1^, and C–H out-plane bending at 1035 cm^−1^ [31,32]. Additionally, the peak at 1166 cm^−1^ is attributed to the pyrrole ring breathing while the peak at 1306 cm^−1^ is attributed to C–N in-plane deformation. Additionally, the stretching vibrations of C–N and C–C bonds were represented by the peaks at 1457 cm^−1^ and 1555 cm^−1^, respectively. It is clear that the ppy chain vibrations were affected by the H-bond formation between NH groups of pyrrole ring and OH groups of NC that caused blue shift of peaks compared to the ppy peaks [33]. To study the crystallinity of the synthesized materials, XRD pattern was performed as shown in Figure 1b. According to Figure 1b, cellulose showed the appearance of major three peaks at angle of 2*θ* = 34.05, 16.45, and 22.84. From the diffractogram of ppy and ppy/NC, there was a slight difference at 2*θ*= 22.84 due to the overlap of ppy peak [34]. Additionally, the cellulose crystalline order was not affected by the hydrolysis reaction using sulfuric acid [35]. The amorphous nature of the nanocomposite was confirmed from the amorphous region at 2*θ* = 22.84 that is related to ppy as indicated from XRD of pure ppy [36]. To study the thermal stability of synthesized materials, thermogravimetric analysis (TGA) of materials was conducted by heating the materials up to 600 °C in argon gas atmosphere as shown in Figure 1c. According to Figure 1c, ppy showed a weight loss of 6% when the temperature raised to 100 °C due to the evaporation of water content. However, the ppy/NC nanocomposite showed weight loss of 4% when the temperature raised to 200 °C due to the evaporation of water residues. At 225 °C, there was a sharp weight loss resulting from thermal decomposition of the pyrolysis of NC. When the temperature was raised to 350 °C, ppy/NC showed a small weight loss due to the decomposition of organic matter of the nanocomposite (cellulose and polypyrrole). Subsequently, the decomposition of organic matter was started above 225 °C and completed at 600 °C (53% wt). The TGA results indicated the high thermal stability of the synthesized ppy/NC nanocomposite. We can conclude that the combination between ppy and cellulose enhances the composite thermal stability. Additionally, Figure 1d showed the zeta potential measurement of the synthesized ppy/NC nanocomposite. According to Figure 1d, the isoelectric point occurred at a value below 6. This point is the zero charge, below which the protonation of nitrogen centers was achieved which eased the attraction between cations and anions.

**Figure 2:**
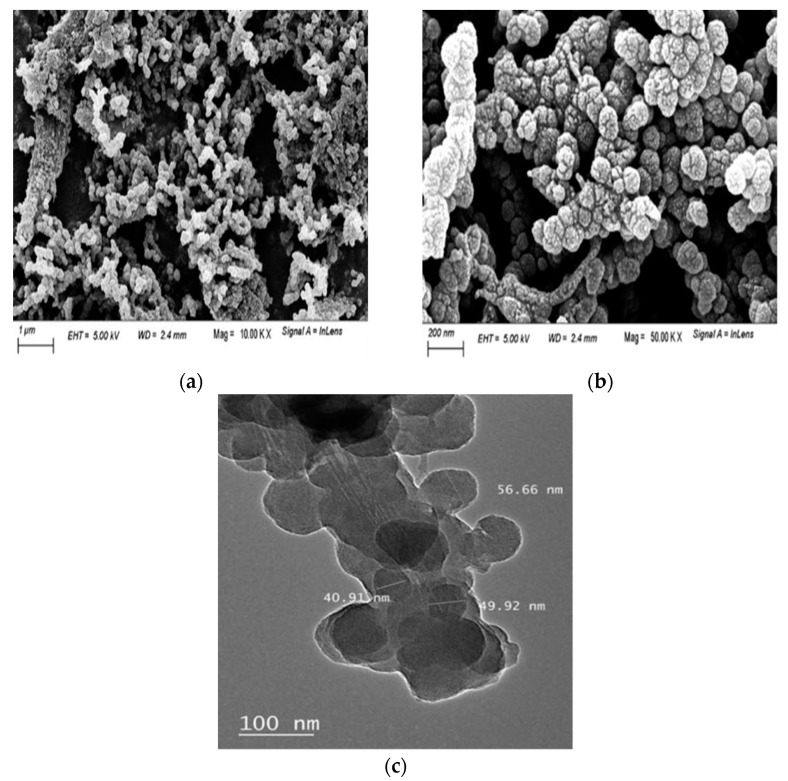
SEM images at different resolution (**a**,**b**) and TEM image (**c**) of the synthesized ppy/NC nanocomposite.

To study the surface morphology of the nanocomposite, SEM images at different magnification as well as TEM images were carried out (Figure 2). Figure S1 (Supplementary Material) showed the SEM image of unmodified nanocellulose with smooth surface. Figure 2a,b showed the SEM images of ppy/NC nanocomposite at different resolutions. SEM images of ppy/NC nanocomposite indicated the homogenous and uniform appearance of the particles with a spherical-like shape in the range of nanometer that help the capture of more toxic metals due to the high surface area. Comparing the SEM images of NC and ppy/NC nanocomposite, the surface of NC was greatly changed due to the introduction of ppy indicating the successful modification of NC surface.

Figure S2 (Supplementary Material) showed the SEM image of ppy/NC nanocomposite after the adsorption of Cr(VI) ions. Figure S2 showed that the surface of the nanocomposite became smooth comparing to the nanocomposite surface due to the capturing of metal ions. Figure 2c showed the TEM image of the nanocomposite. According to Figure 2d, the nanoparticles of nanocellulose are agglomerated in some parts and separated in the others. Additionally, there is a major uniform distribution of the nanocellulose particles in the nanometer range. As concluded from the TEM image, the average size of nanocellulose particles is 40 nm to 50 nm.

### 3.2. Effect of pH on Cr(VI) Adsorption

**Figure 3:**
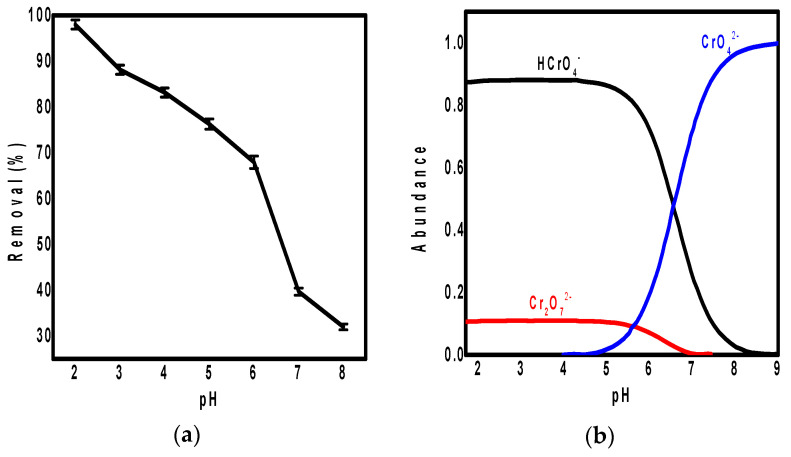
The effect of pH on Cr(VI) adsorption on the ppy/NC surface (**a**) and the abundance of Cr(VI) species in aqueous solution at pH range of 2 to 9 (**b**).

The pH value of solution is a critical factor affecting the adsorption behavior [37] specially for Cr(VI) ions. Therefore, the pH effect on the adsorption of Cr(VI) on the surface of ppy/NC nanocomposite was studied at pH ranging from 2 to 8 as shown in Figure 3. According to Figure 3a, the ppy/NC nanocomposite showed the highest removal efficiency for Cr(VI) ions at pH value of 2 (98%) while this removal efficiency was decreased gradually by increasing pH value until reached its lowest efficiency at pH 8 (32%). These results of pH effect on the adsorption could be interpreted according to the ionic behavior of chromium ions at different pH as illustrated in Figure 3b.

According to Figure 3b, Cr_2_O_7_^2−^ and HCrO_4_^−^ are the two oxidation states of chromium ions in the pH range of 2 to 6 while at higher pH the chromium phase becomes CrO_4_^2−^ [38]. Subsequently, at pH between 2 and 6, the adsorption of Cr(VI) ions exists from the electrostatic attraction between the positively charged adsorbent and the negatively charged chromium species. Additionally, the functionalization of nano-cellulose with polypyrrole helps the ion exchange between the adsorbent and chromium species that increases the adsorption efficiency. However, at the pH value > 6, there was a great competition between the OH^−^ ions and negatively charged chromium species for the adsorption sites on the adsorbent surface decreasing the adsorption efficiency. From the study of the pH effect, we can conclude that the pH increase changed the adsorption of Cr(VI) ions on the surface of ppy/NC nanocomposite from the electrostatic attraction to the electrostatic repulsion. In other words, the adsorption was changed from promotion to competition which would cause the decrease of the removal efficiency.

### 3.3. Effect of Co-Existing Ions on Cr(VI) Removal

**Figure 4:**
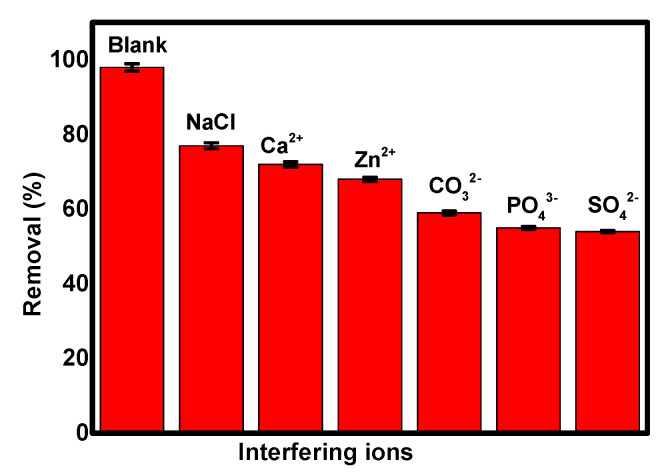
The effect of interfering ions on the Cr(VI) removal over the surface of ppy/NC nanocomposite.

Different ions such as NaCl, Ca^2+^, Zn^2+^, CO_3_^2−^, PO_4_^3−^, and SO_4_^2−^ may exist in real wastewater and could affect the adsorption of Cr(VI) ions [39,40]. So, the effect of different co-existing ions on the adsorption of Cr(VI) ions on the surface of ppy/NC nanocomposite was studied in the presence of 0.1 M of each ion as shown in Figure 4.

According to Figure 4, the removal efficiency of Cr(VI) ions was 98% in the absence of any interfering ions. Then, this removal efficiency was decreased on the addition of different ions in the order of NaCl > Ca^2+^ > Zn^2+^ > CO_3_^2−^ > PO_4_^3−^ > SO_4_^2−^ with removal efficiencies of 77%, 72%, 68%, 59%, 55%, and 54%, respectively. This decrease in the removal efficiency of Cr(VI) ions was attributed to the competition of interfering ions with chromium ions for the adsorption sites on the adsorbent surface. This order of removal efficiencies is attributed to the differences in the solvated ionic radii between the co-existing ions. Generally, small ions have the ability to reach the adsorption sites easily and compete with toxic metals leading to the drop of the removal efficiency of this metal. According to these results, the ppy/NC nanocomposite can be efficiently used for the removal of hexavalent chromium ions even in the presence of interfering ions.

### 3.4. Adsorption Kinetics

**Figure 5:**
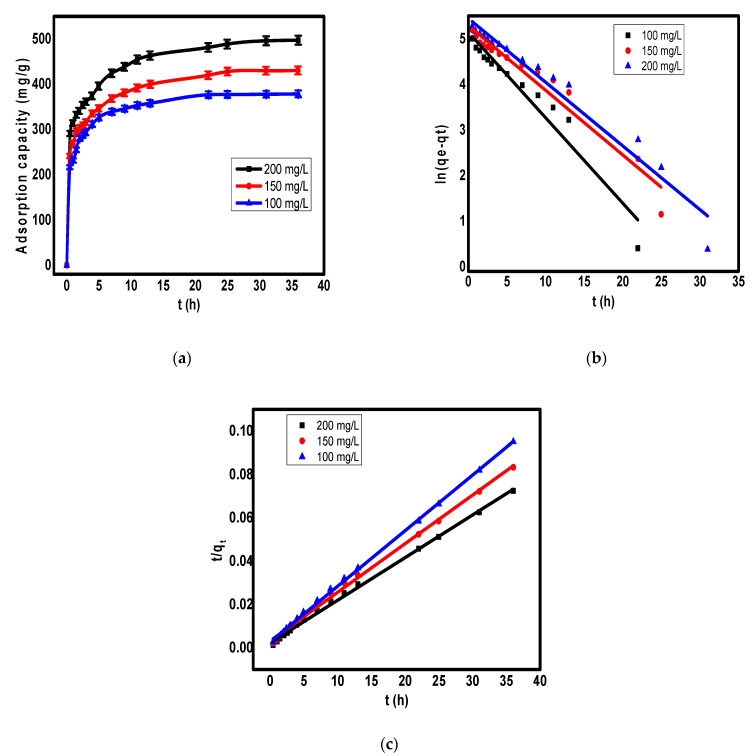
The effect of contact time at different initial Cr(VI) concentrations (**a**), fit of experimental data to pseudo 1st order model (**b**), and fit of experimental data to pseudo 2nd order model (**c**) for the removal of Cr(VI) ions on the surface of ppy/NC nanocomposite.

To study the contact time effect on the adsorption of Cr(VI) on the surface of ppy/NC nanocomposite, the adsorption capacities were studied at different time intervals from 0 to 35 h using different initial concentrations of chromium ions as shown in Figure 5a. According to Figure 5a, the first 3 h showed a rapid increase of the adsorption capacity followed by a slow increase of this capacity until it reached the equilibrium. The rapid increase of adsorption capacity during the first hours could be attributed to the availability of large number of active sites for the uptake of toxic ions [41]. After that, there was a slow increase in the adsorption capacity due to the repulsion between already adsorbed ions on the adsorbent and the other ions until reach the equilibrium at which the adsorbent surface becomes saturated, and the time does not have a significant effect on the adsorption capacity.

The two kinetic models pseudo 1st order and pseudo 2nd order were used to study the adsorption kinetics of Cr(VI) ions on the surface of ppy/NC nanocomposite. The linear form of pseudo 1st order and pseudo 2nd order is given by Equations (3) and (4), respectively.

ln (q_e_ − q_t_) = ln q_e_ − k_1_t (3)

t/q_t_ = (1/k_2_q_e_^2^) + t/q_e_ (4)

where k_1_ (1/min) and k_2_ (g/mg·min) are the rate constants for pseudo 1st order model and pseudo 2nd order model, respectively. The two constants can be calculated from the slop and intercept of the Equations (3) and (4) plots as shown in Figure 5b,c. The kinetic parameters for Cr(VI) removal on the surface of ppy/NC were listed in Table 1.

According to data in Table 1, the correlation coefficient (R^2^) value indicated that the adsorption kinetic data were more fitted with the pseudo 2nd order model than the pseudo 1st order model. This means that the pseudo 2nd order model is more suitable to describe the adsorption mechanism of Cr(VI) ions on the surface of ppy/NC nanocomposite. These results indicated that the chemical sorption is the essential mechanism of Cr(VI) removal on the surface of ppy/NC nanocomposite rather than a usual mass transport [42,43]. Additionally, several previous works on the adsorption of Cr(VI) ions showed the applicability of pseudo 2nd order as a better model for the description of the removal mechanism than other kinetic models [44,45,46,47].

### 3.5. Adsorption Isotherm

The interaction between the adsorbent surface and toxic particles can be well-interpreted by the study of the adsorption isotherms. The two isotherms models Freundlich and Langmuir were used to study the experimental data of Cr(VI) removal on the surface of ppy/NC nanocomposite. Freundlich and Langmuir isotherms can be described in the linearized form according to Equations (5) and (6), respectively.

ln q_e_ = ln K_F_ + 1/n ln C_e_, (5)

**Figure 6:**
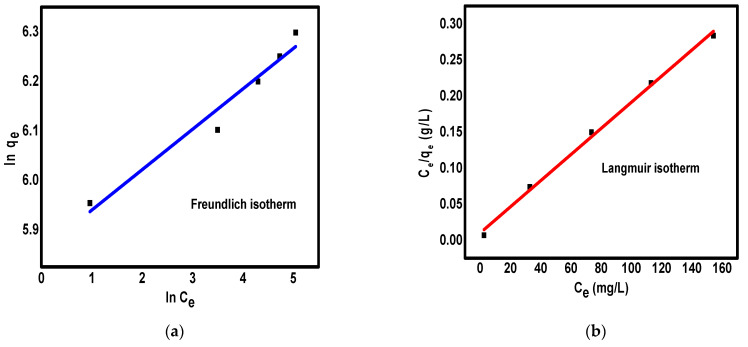
The fit of experimental data to Freundlich isotherm (**a**) and Langmuir isotherm (**b**) for the removal of Cr(VI) ions on the surface of ppy/NC nanocomposite.

1/q_e_ = (1/q_m_k_L_)C_e_ + 1/q_m_, (6)

where 1/n, K_F_, K_L_, and q_m_ denote the adsorption intensity, Freundlich constant, Langmuir constant, and the maximum adsorption capacity, respectively. It is well-known that Freundlich isotherm suggests the exponential distribution of the adsorption sites as well as the heterogeneous properties of the surface while the Langmuir isotherm suggests the presence of finite amount of identical adsorption sites on the surface of adsorbent as well as the formation of monolayer of adsorbates [48,49]. The fitting of experimental data to Freundlich and Langmuir isotherm models are shown in Figure 6 and their parameters were tabulated in Table 2. The Freundlich and Langmuir parameters were calculated by the plot of ln q_e_ against ln C_e_ and C_e_/q_e_ against C_e_, respectively. According to the results, 1/n value is less than 1 indicating the favorable adsorption of Cr(VI) on the surface of ppy/NC nanocomposite [50,51].

The values of correlation coefficients (R^2^) were 0.948 for Freundlich isotherm and 0.997 for Langmuir isotherm indicated that the adsorption of Cr(VI) can be better defined by the Langmuir isotherm model. The isotherm results indicated that the capture of Cr(VI) ions on the surface of ppy/NC nanocomposite was reached via a monolayer adsorption by identical adsorption active sites [52,53]. Moreover, the maximum adsorption capacity of the nanocomposite was reached 560 mg/L toward Cr(VI) ions that is considered excellent results when compared to other adsorbents as discussed in the comparative study section.

### 3.6. Discussion on the Mechanism of Removal

**Figure 7:**
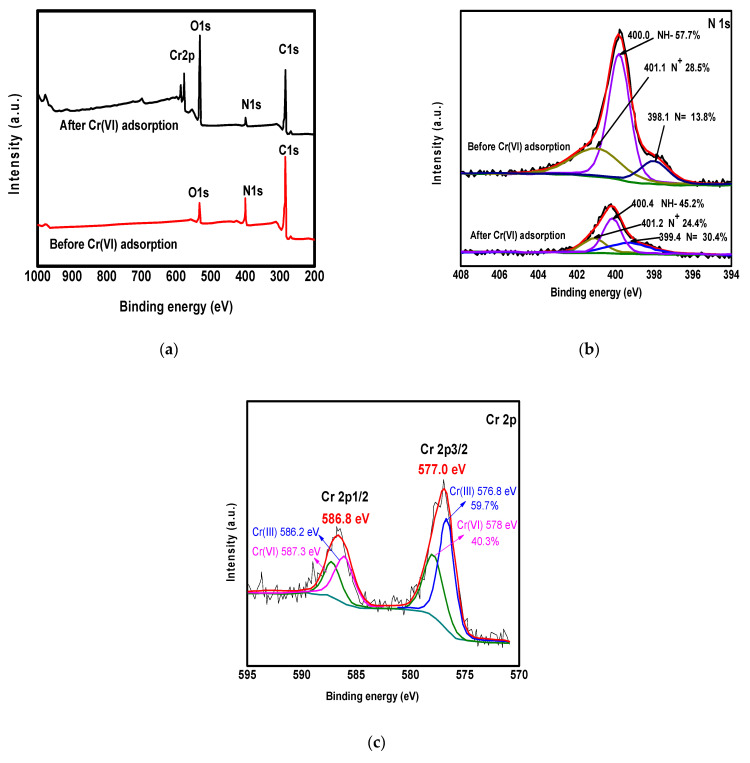
XPS spectra of ppy/NC nanocomposite with survey scan (**a**), N 1s spectra (**b**), and (**c**) Cr 2p spectra before and after adsorption process.

To understand the mechanism of the removal of Cr(VI) ions on the surface of ppy/NC nanocomposite very well, XPS spectra of the nanocomposite before and after adsorption are reported in Figure 7. Figure 7a shows the scan survey of the nanocomposite. According to Figure 7a, the C 1s, N1s, and O 1s peaks appeared at binding energies of 285.0 eV, 400.0 eV, and 532.4, respectively [54]. A peak related to Cr 2p was appeared after the capturing process indicated the uptake of the metal. This peak appeared as a twin attributed to Cr 2p_3/2_ and Cr 2p_1/2_ at 577.0 and 586.9 eV, respectively. The oxygen content of the adsorbed chromium ions made the C/O ratio of the composite increased considerably after the adsorption as shown in the O 1s peak. Additionally, the N 1s spectra of the nanocomposite were completed before and after the metal uptake as shown in Figure 7b.

According to Figure 7b, N^+^, NH-, and N- peaks were observed with molar ratios equal to 13.8, 28.5, and 57.7%, respectively. These ratios by the action of strong oxidation of Cr(VI) were increased to 30.4, 24.4, and 45.2%, respectively. Moreover, the Cr 2p spectra of the nanocomposite were performed to explain the mechanism of metal removal deeply as shown in Figure 7c. After the adsorption, the nanocomposite showed the appearance of Cr (III) and Cr (VI) peaks with molar ratios of 59.7% and 40.3%, respectively, meaning that the ppy electrons reduces the hexavalent chromium to the trivalent chromium and the adsorption process included both ions. This removal mechanism was described by many previous studies [55,56,57,58].

### 3.7. Reusability Study

**Figure 8:**
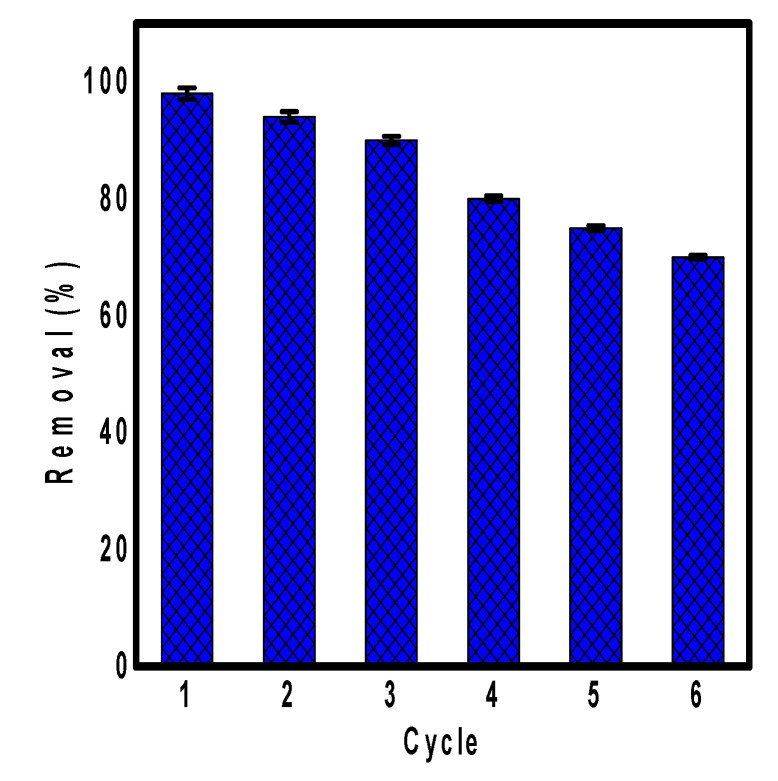
The reusability study of ppy/NC nanocomposite for the removal of Cr(VI) ions up to six successive cycles.

For the large-scale application of any adsorbent, its reusability and regeneration must be studied as it is very important economically, and a good reusability may reduce the overall cost of treatment [59]. Subsequently, the reusability of ppy/NC nanocomposite for the removal of Cr(VI) ions was studied for six successive cycles as shown in Figure 8. Each cycle is consisting of adsorption process followed by desorption process. The adsorbent was agitated with the metallic ion solution followed by the treatment of the adsorbent surface with 1 M of HCl as eluent. Finally, the adsorbent became ready for the next cycle.

According to Figure 8, the ppy/NC nanocomposite showed good reusability results for the removal of Cr(VI) ions up to six cycles. The first cycle showed the highest removal efficiency due to the existence of fresh unused adsorption sites on the surface of adsorbent. After that, the removal efficiency was decreased gradually due to the destroyed non-renewable sites during each cycle. The last cycle showed a removal efficiency of 70% indicated the excellent stability of the nanocomposite toward the removal of Cr(VI) ions. These results indicated the effective regeneration of adsorption sites during the adsorption process which reduces the overall cost of process and encourages the application of the nanocomposite for large scale treatment.

### 3.8. Comparative Study

To assess the performance of ppy/NC as an adsorbent for the removal of Cr(VI) from aqueous solution, the adsorption capacities of previously reported adsorbents were compared with the present study as listed in Table 3. According to Table 3, the ppy/NC showed an excellent adsorption capacity toward Cr(VI) ions. This good capacity encourages its use as adsorbent for real samples treatment. Additionally, this adsorbent could be examined for the removal of many other pollutants.

## 4. Conclusions

In the present study, cellulose was hydrolyzed using sulfuric acid to produce the nanocellulose, and then functionalized using polypyrrole through a polymerization reaction to fabricate a nanocomposite of polypyrrole/nanocellulose (ppy/NC). The familiar techniques were used for the characterization of the nanocomposite. The crystalline nature of the synthesized NC was approved using XRD. Additionally, the nanosized cellulose was approved through SEM images. Thermal analysis study showed the excellent stability of the nanocomposite with a decomposition point at 225 °C. The synthesized nanocomposite was examined for the adsorption of Cr(VI) ions. The pH effect on the adsorption of metallic ions was studied showing an optimum at pH 2.0. Additionally, the effect of interfering ions on the removal efficiency (%) was studied with minor decrease in the Cr(VI) removal due to the competition between ions for the adsorption sites. The nanocomposite showed a maximum adsorption capacity of 560 mg/g according to Langmuir isotherm model. The study of the removal mechanism showed that the hexavalent chromium was removed via two ways of adsorption and reduction which enhanced the performance of the adsorbent. Additionally, the ppy/NC adsorbent was reused for the removal of Cr(VI) ions up to six cycles with excellent regeneration results. The comparative study of ppy/NC nanocomposite with other reported adsorbents confirmed its higher performance for Cr(VI) uptake. Hopefully, this nanocomposite is an excellent choice for water treatment and should be investigated toward additional pollutants in the near future.

## Figures and Tables

**Table 1:** The parameters of pseudo 1st order and pseudo 2nd order models for the removal of Cr(VI) on the surface of ppy/NC nanocomposite.

Cr(VI) Concentration (mg/L)	Pseudo 1st Order				Pseudo 2nd Order		
	q_e_(exp) (mg/g)	q_e_(cal) (mg/g)	K_1_	R^2^	q_e_(cal) (mg/g)	K_2_	R^2^
100	379	172	0.1880	0.9388	385.1	0.0031	0.9993
150	432.1	199	0.1433	0.9499	443.2	0.0019	0.9988
200	496.2	233.2	0.1395	0.9499	508.6	0.0018	0.9979

**Table 2:** The parameters of Freundlish and Langmuir isotherm models for the removal of Cr(VI) on the surface of ppy/NC nanocomposite.

Langmuir			Freundlich		
q_m_ (mg/g)	K_L_	R^2^	K_F_	1/n	R^2^
560	0.2300	0.997	350.1	0.0941	0.949

**Table 3:** The comparison between the removal of Cr(VI) ions on the surface of ppy/NC nanocomposite and reported studies.

Adsorbent	Adsorption Capacity (mg/g)	Ref.
ppy/NC	560.0	This study
Chitosan-crosslinked-poly(alginic acid) nanohydrogel	26.42	[60]
Activated carbon from peanut shell	16.27	[61]
Polyaniline-coated electrospun adsorbent membrane	15.09	[62]
Biochar modified with Mg/Al-layered double hydroxide intercalated with EDTA	38.0	[63]
Sulfuric acid modified leaves	107.55	[64]
Graphene/SiO_2_@PPy nanocomposites	429.0	[65]
PA6@Mg(OH)_2_ electrospun nanofibers	296.0	[66]
PAN-NH_2_ nanofibers	138.0	[67]
PAN/polypyrrole core/shell nanofiber mat	75.0	[68]
Ammonium-functionalized cellulose nanofibers	18.0	[69]

## Data Availability

Not applicable.

## Supplementary Materials

The following are available online at www.mdpi.com/2073-4360/13/21/3691/s1, Figure S1: SEM image of nanocellulose, Figure S2: SEM image of ppy/NC nanocomposite after Cr(VI) ions adsorption.
