# Supplementary material for: Synthesis, Characterization and Application of Polypyrrole Functionalized Nanocellulose for the Removal of Cr(VI) from Aqueous Solution
Source: Polymers (Basel). 2021 Oct 26;13(21):3691. doi: 10.3390/polym13213691 (PMC8587301; doi:10.3390/polym13213691)
Supplement: Supplementary file 1 [file polymers-13-03691-s001.zip › polymers-1432448-supplementary.pdf]

Supplementary Material

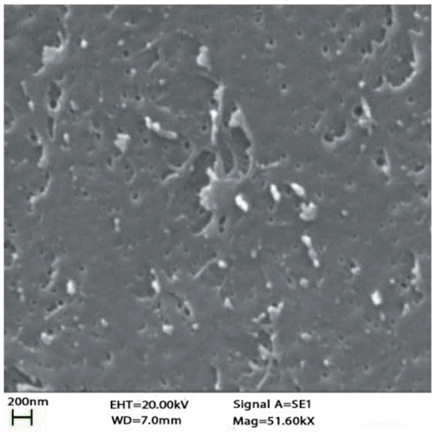

Figure S1. SEM image of nanocellulose.

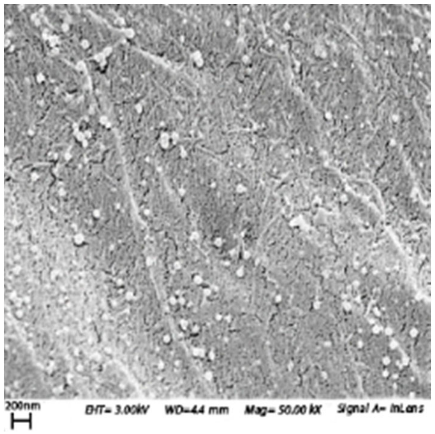

Figure S2. SEM image of ppy/NC nanocomposite after Cr(VI) ions adsorption.
